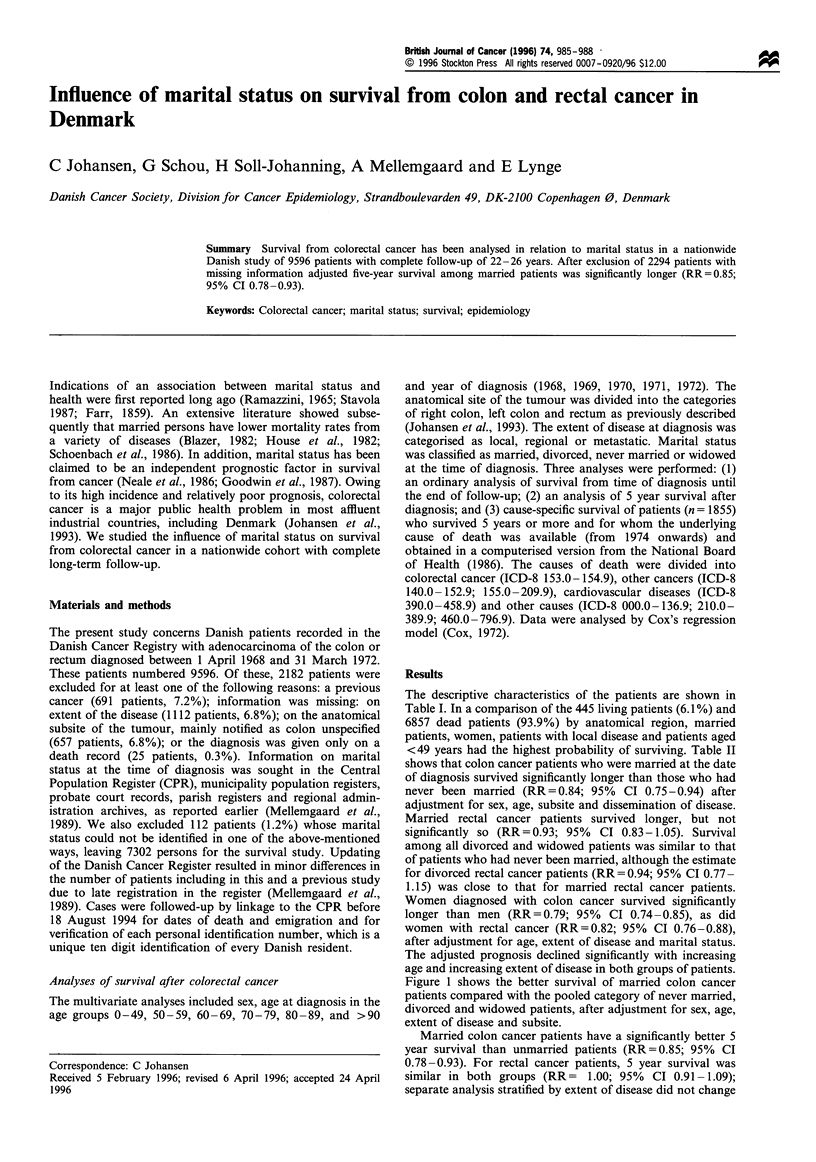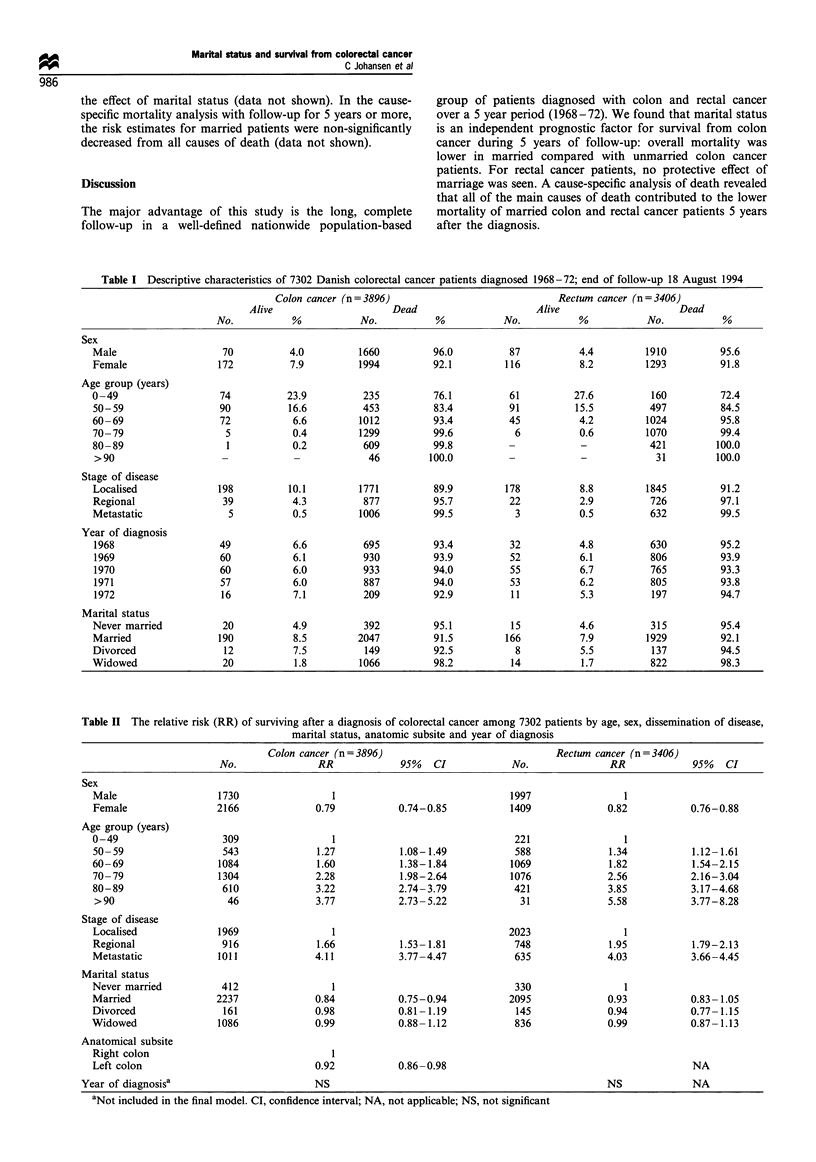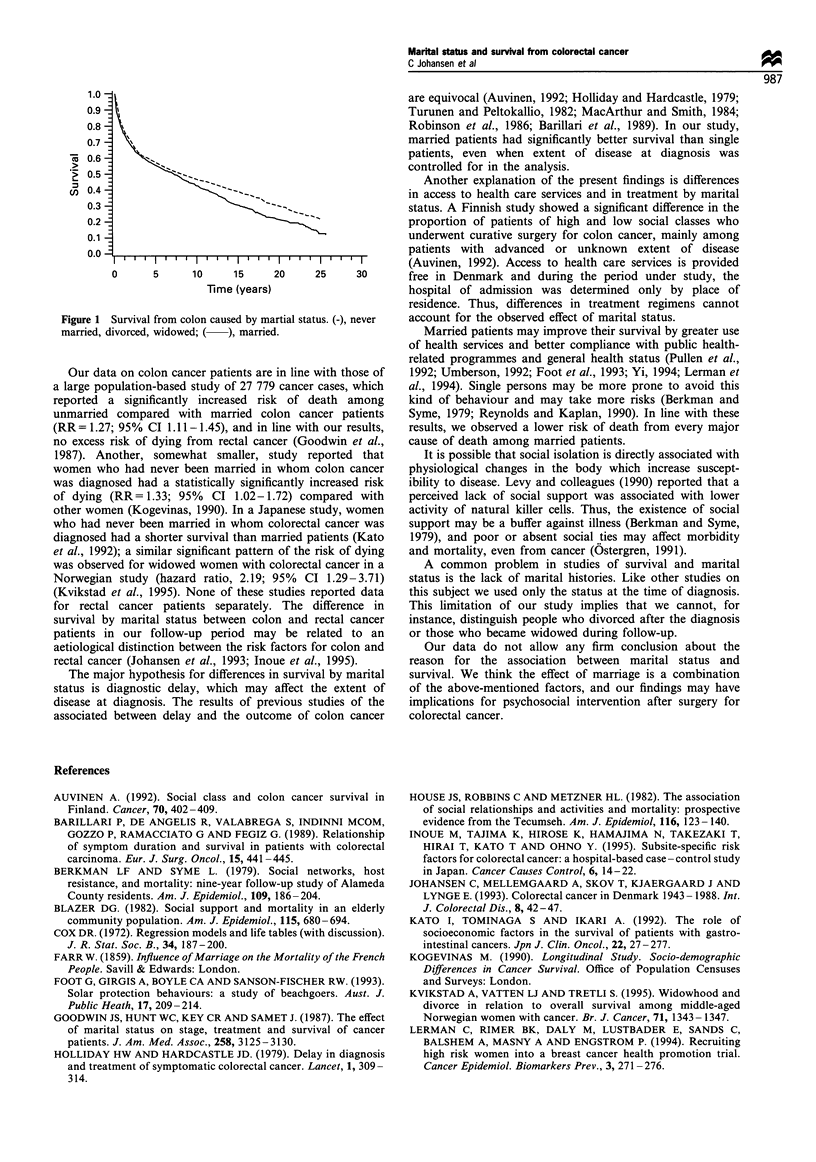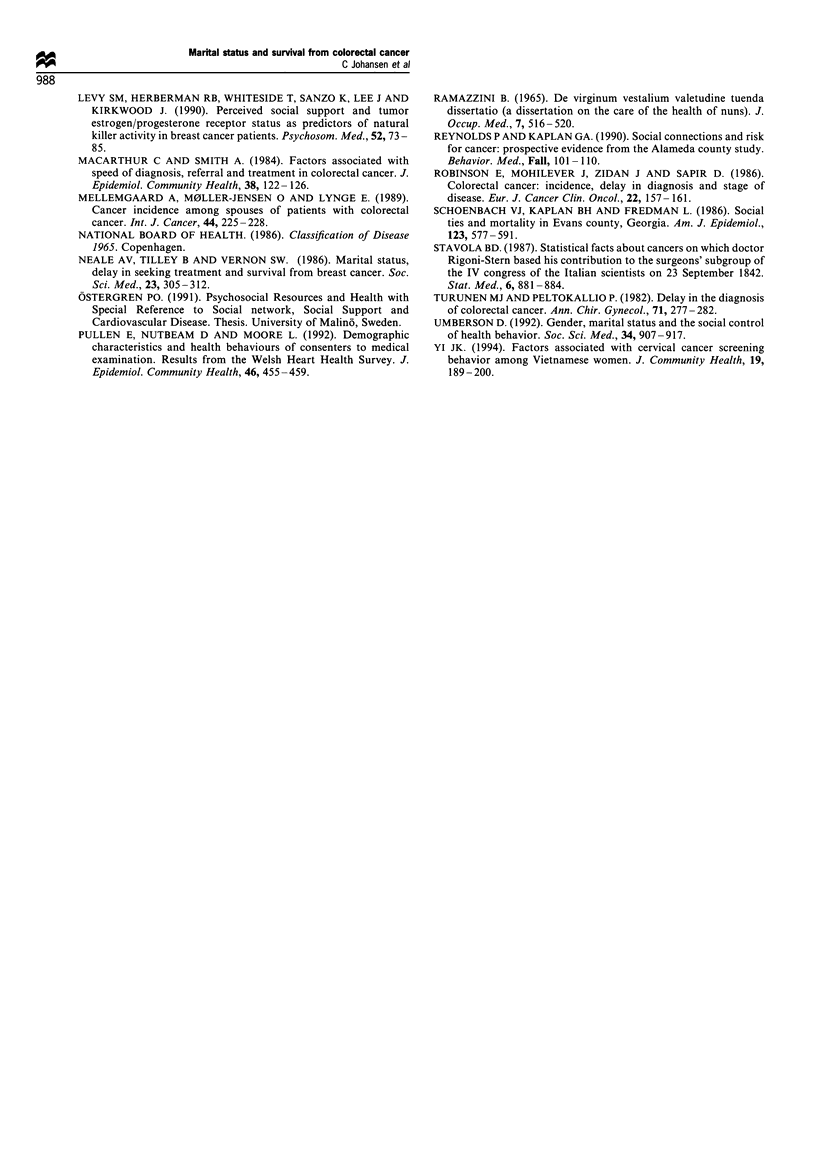# Influence of marital status on survival from colon and rectal cancer in Denmark

**DOI:** 10.1038/bjc.1996.470

**Published:** 1996-09

**Authors:** C Johansen, G Schou, H Soll-Johanning, A Mellemgaard, E Lynge

**Keywords:** Colorectal cancer, marital status, survival, epidemiology

## Abstract

Survival from colorectal cancer has been analysed in relation to marital status in a nationwide Danish study of 9596 patients with complete follow-up of 22-26 years. After exclusion of 2294 patients with missing information adjusted five-year survival among married patients was significantly longer (RR=0.85; 95% CI 0.78-0.93).


British Journal of Cancer (1996) 74, 985-988
© 1996 Stockton Press All rights reserved 0007-0920/96 $12.00

### Influence of marital status on survival from colon and rectal cancer in Denmark

C Johansen, G Schou, H Soll-Johanning, A Mellemgaard and E Lynge

*Danish Cancer Society, Division for Cancer Epidemiology, Strandboulevarden 49, DK-2100 Copenhagen Ø, Denmark*

**Summary** Survival from colorectal cancer has been analysed in relation to marital status in a nationwide Danish study of 9596 patients with complete follow-up of 22-26 years. After exclusion of 2294 patients with missing information adjusted five-year survival among married patients was significantly longer (RR=0.85; 95% CI 0.78-0.93).

**Keywords:** Colorectal cancer; marital status; survival; epidemiology

Indications of an association between marital status and health were first reported long ago (Ramazzini, 1965; Stavola 1987; Farr, 1859). An extensive literature showed subsequently that married persons have lower mortality rates from a variety of diseases (Blazer, 1982; House *et al.*, 1982; Schoenbach *et al.*, 1986). In addition, marital status has been claimed to be an independent prognostic factor in survival from cancer (Neale *et al.*, 1986; Goodwin *et al.*, 1987). Owing to its high incidence and relatively poor prognosis, colorectal cancer is a major public health problem in most affluent industrial countries, including Denmark (Johansen *et al.*, 1993). We studied the influence of marital status on survival from colorectal cancer in a nationwide cohort with complete long-term follow-up.

### Materials and methods

The present study concerns Danish patients recorded in the Danish Cancer Registry with adenocarcinoma of the colon or rectum diagnosed between 1 April 1968 and 31 March 1972. These patients numbered 9596. Of these, 2182 patients were excluded for at least one of the following reasons: a previous cancer (691 patients, 7.2%); information was missing: on extent of the disease (1112 patients, 6.8%); on the anatomical subsite of the tumour, mainly notified as colon unspecified (657 patients, 6.8%); or the diagnosis was given only on a death record (25 patients, 0.3%). Information on marital status at the time of diagnosis was sought in the Central Population Register (CPR), municipality population registers, probate court records, parish registers and regional administration archives, as reported earlier (Mellemgaard *et al.*, 1989). We also excluded 112 patients (1.2%) whose marital status could not be identified in one of the above-mentioned ways, leaving 7302 persons for the survival study. Updating of the Danish Cancer Register resulted in minor differences in the number of patients including in this and a previous study due to late registration in the register (Mellemgaard *et al.*, 1989). Cases were followed-up by linkage to the CPR before 18 August 1994 for dates of death and emigration and for verification of each personal identification number, which is a unique ten digit identification of every Danish resident.

### *Analyses of survival after colorectal cancer*

The multivariate analyses included sex, age at diagnosis in the age groups 0-49, 50-59, 60-69, 70-79, 80-89, and >90 and year of diagnosis (1968, 1969, 1970, 1971, 1972). The anatomical site of the tumour was divided into the categories of right colon, left colon and rectum as previously described (Johansen *et al.*, 1993). The extent of disease at diagnosis was categorised as local, regional or metastatic. Marital status was classified as married, divorced, never married or widowed at the time of diagnosis. Three analyses were performed: (1) an ordinary analysis of survival from time of diagnosis until the end of follow-up; (2) an analysis of 5 year survival after diagnosis; and (3) cause-specific survival of patients ($n=1855$) who survived 5 years or more and for whom the underlying cause of death was available (from 1974 onwards) and obtained in a computerised version from the National Board of Health (1986). The causes of death were divided into colorectal cancer (ICD-8 153.0-154.9), other cancers (ICD-8 140.0-152.9; 155.0-209.9), cardiovascular diseases (ICD-8 390.0-458.9) and other causes (ICD-8 000.0-136.9; 210.0-389.9; 460.0-796.9). Data were analysed by Cox's regression model (Cox, 1972).

### Results

The descriptive characteristics of the patients are shown in Table I. In a comparison of the 445 living patients (6.1%) and 6857 dead patients (93.9%) by anatomical region, married patients, women, patients with local disease and patients aged <49 years had the highest probability of surviving. Table II shows that colon cancer patients who were married at the date of diagnosis survived significantly longer than those who had never been married (RR=0.84; 95% CI 0.75-0.94) after adjustment for sex, age, subsite and dissemination of disease. Married rectal cancer patients survived longer, but not significantly so (RR=0.93; 95% CI 0.83-1.05). Survival among all divorced and widowed patients was similar to that of patients who had never been married, although the estimate for divorced rectal cancer patients (RR=0.94; 95% CI 0.77-1.15) was close to that for married rectal cancer patients. Women diagnosed with colon cancer survived significantly longer than men (RR=0.79; 95% CI 0.74-0.85), as did women with rectal cancer (RR=0.82; 95% CI 0.76-0.88), after adjustment for age, extent of disease and marital status. The adjusted prognosis declined significantly with increasing age and increasing extent of disease in both groups of patients. Figure 1 shows the better survival of married colon cancer patients compared with the pooled category of never married, divorced and widowed patients, after adjustment for sex, age, extent of disease and subsite.

Married colon cancer patients have a significantly better 5 year survival than unmarried patients (RR=0.85; 95% CI 0.78-0.93). For rectal cancer patients, 5 year survival was similar in both groups (RR= 1.00; 95% CI 0.91-1.09); separate analysis stratified by extent of disease did not change

Correspondence: C Johansen
Received 5 February 1996; revised 6 April 1996; accepted 24 April 1996

the effect of marital status (data not shown). In the cause-specific mortality analysis with follow-up for 5 years or more, the risk estimates for married patients were non-significantly decreased from all causes of death (data not shown).

### Discussion

The major advantage of this study is the long, complete follow-up in a well-defined nationwide population-based group of patients diagnosed with colon and rectal cancer over a 5 year period (1968–72). We found that marital status is an independent prognostic factor for survival from colon cancer during 5 years of follow-up: overall mortality was lower in married compared with unmarried colon cancer patients. For rectal cancer patients, no protective effect of marriage was seen. A cause-specific analysis of death revealed that all of the main causes of death contributed to the lower mortality of married colon and rectal cancer patients 5 years after the diagnosis.

**Table I** Descriptive characteristics of 7302 Danish colorectal cancer patients diagnosed 1968–72; end of follow-up 18 August 1994

| | *Colon cancer* (n=*3896*) | | | | *Rectum cancer* (n=*3406*) | | | |
|---|---|---|---|---|---|---|---|---|
| | *Alive* | | *Dead* | | *Alive* | | *Dead* | |
| | *No.* | *%* | *No.* | *%* | *No.* | *%* | *No.* | *%* |
| Sex | | | | | | | | |
| Male | 70 | 4.0 | 1660 | 96.0 | 87 | 4.4 | 1910 | 95.6 |
| Female | 172 | 7.9 | 1994 | 92.1 | 116 | 8.2 | 1293 | 91.8 |
| Age group (years) | | | | | | | | |
| 0–49 | 74 | 23.9 | 235 | 76.1 | 61 | 27.6 | 160 | 72.4 |
| 50–59 | 90 | 16.6 | 453 | 83.4 | 91 | 15.5 | 497 | 84.5 |
| 60–69 | 72 | 6.6 | 1012 | 93.4 | 45 | 4.2 | 1024 | 95.8 |
| 70–79 | 5 | 0.4 | 1299 | 99.6 | 6 | 0.6 | 1070 | 99.4 |
| 80–89 | 1 | 0.2 | 609 | 99.8 | – | – | 421 | 100.0 |
| >90 | – | – | 46 | 100.0 | – | – | 31 | 100.0 |
| Stage of disease | | | | | | | | |
| Localised | 198 | 10.1 | 1771 | 89.9 | 178 | 8.8 | 1845 | 91.2 |
| Regional | 39 | 4.3 | 877 | 95.7 | 22 | 2.9 | 726 | 97.1 |
| Metastatic | 5 | 0.5 | 1006 | 99.5 | 3 | 0.5 | 632 | 99.5 |
| Year of diagnosis | | | | | | | | |
| 1968 | 49 | 6.6 | 695 | 93.4 | 32 | 4.8 | 630 | 95.2 |
| 1969 | 60 | 6.1 | 930 | 93.9 | 52 | 6.1 | 806 | 93.9 |
| 1970 | 60 | 6.0 | 933 | 94.0 | 55 | 6.7 | 765 | 93.3 |
| 1971 | 57 | 6.0 | 887 | 94.0 | 53 | 6.2 | 805 | 93.8 |
| 1972 | 16 | 7.1 | 209 | 92.9 | 11 | 5.3 | 197 | 94.7 |
| Marital status | | | | | | | | |
| Never married | 20 | 4.9 | 392 | 95.1 | 15 | 4.6 | 315 | 95.4 |
| Married | 190 | 8.5 | 2047 | 91.5 | 166 | 7.9 | 1929 | 92.1 |
| Divorced | 12 | 7.5 | 149 | 92.5 | 8 | 5.5 | 137 | 94.5 |
| Widowed | 20 | 1.8 | 1066 | 98.2 | 14 | 1.7 | 822 | 98.3 |

**Table II** The relative risk (RR) of surviving after a diagnosis of colorectal cancer among 7302 patients by age, sex, dissemination of disease, marital status, anatomic subsite and year of diagnosis

| | *Colon cancer* (n=*3896*) | | | *Rectum cancer* (n=*3406*) | | |
|---|---|---|---|---|---|---|
| | *No.* | *RR* | *95% CI* | *No.* | *RR* | *95% CI* |
| Sex | | | | | | |
| Male | 1730 | 1 | | 1997 | 1 | |
| Female | 2166 | 0.79 | 0.74–0.85 | 1409 | 0.82 | 0.76–0.88 |
| Age group (years) | | | | | | |
| 0–49 | 309 | 1 | | 221 | 1 | |
| 50–59 | 543 | 1.27 | 1.08–1.49 | 588 | 1.34 | 1.12–1.61 |
| 60–69 | 1084 | 1.60 | 1.38–1.84 | 1069 | 1.82 | 1.54–2.15 |
| 70–79 | 1304 | 2.28 | 1.98–2.64 | 1076 | 2.56 | 2.16–3.04 |
| 80–89 | 610 | 3.22 | 2.74–3.79 | 421 | 3.85 | 3.17–4.68 |
| >90 | 46 | 3.77 | 2.73–5.22 | 31 | 5.58 | 3.77–8.28 |
| Stage of disease | | | | | | |
| Localised | 1969 | 1 | | 2023 | 1 | |
| Regional | 916 | 1.66 | 1.53–1.81 | 748 | 1.95 | 1.79–2.13 |
| Metastatic | 1011 | 4.11 | 3.77–4.47 | 635 | 4.03 | 3.66–4.45 |
| Marital status | | | | | | |
| Never married | 412 | 1 | | 330 | 1 | |
| Married | 2237 | 0.84 | 0.75–0.94 | 2095 | 0.93 | 0.83–1.05 |
| Divorced | 161 | 0.98 | 0.81–1.19 | 145 | 0.94 | 0.77–1.15 |
| Widowed | 1086 | 0.99 | 0.88–1.12 | 836 | 0.99 | 0.87–1.13 |
| Anatomical subsite | | | | | | |
| Right colon | | 1 | | | | |
| Left colon | | 0.92 | 0.86–0.98 | | | NA |
| Year of diagnosis[a] | | NS | | | NS | NA |

[a]Not included in the final model. CI, confidence interval; NA, not applicable; NS, not significant

**Figure 1** Survival from colon caused by martial status. (-), never married, divorced, widowed; (——), married.

Our data on colon cancer patients are in line with those of a large population-based study of 27 779 cancer cases, which reported a significantly increased risk of death among unmarried compared with married colon cancer patients (RR = 1.27; 95% CI 1.11 – 1.45), and in line with our results, no excess risk of dying from rectal cancer (Goodwin *et al.*, 1987). Another, somewhat smaller, study reported that women who had never been married in whom colon cancer was diagnosed had a statistically significantly increased risk of dying (RR = 1.33; 95% CI 1.02 – 1.72) compared with other women (Kogevinas, 1990). In a Japanese study, women who had never been married in whom colorectal cancer was diagnosed had a shorter survival than married patients (Kato *et al.*, 1992); a similar significant pattern of the risk of dying was observed for widowed women with colorectal cancer in a Norwegian study (hazard ratio, 2.19; 95% CI 1.29 – 3.71) (Kvikstad *et al.*, 1995). None of these studies reported data for rectal cancer patients separately. The difference in survival by marital status between colon and rectal cancer patients in our follow-up period may be related to an aetiological distinction between the risk factors for colon and rectal cancer (Johansen *et al.*, 1993; Inoue *et al.*, 1995).

The major hypothesis for differences in survival by marital status is diagnostic delay, which may affect the extent of disease at diagnosis. The results of previous studies of the associated between delay and the outcome of colon cancer are equivocal (Auvinen, 1992; Holliday and Hardcastle, 1979; Turunen and Peltokallio, 1982; MacArthur and Smith, 1984; Robinson *et al.*, 1986; Barillari *et al.*, 1989). In our study, married patients had significantly better survival than single patients, even when extent of disease at diagnosis was controlled for in the analysis.

Another explanation of the present findings is differences in access to health care services and in treatment by marital status. A Finnish study showed a significant difference in the proportion of patients of high and low social classes who underwent curative surgery for colon cancer, mainly among patients with advanced or unknown extent of disease (Auvinen, 1992). Access to health care services is provided free in Denmark and during the period under study, the hospital of admission was determined only by place of residence. Thus, differences in treatment regimens cannot account for the observed effect of marital status.

Married patients may improve their survival by greater use of health services and better compliance with public health-related programmes and general health status (Pullen *et al.*, 1992; Umberson, 1992; Foot *et al.*, 1993; Yi, 1994; Lerman *et al.*, 1994). Single persons may be more prone to avoid this kind of behaviour and may take more risks (Berkman and Syme, 1979; Reynolds and Kaplan, 1990). In line with these results, we observed a lower risk of death from every major cause of death among married patients.

It is possible that social isolation is directly associated with physiological changes in the body which increase susceptibility to disease. Levy and colleagues (1990) reported that a perceived lack of social support was associated with lower activity of natural killer cells. Thus, the existence of social support may be a buffer against illness (Berkman and Syme, 1979), and poor or absent social ties may affect morbidity and mortality, even from cancer (Östergren, 1991).

A common problem in studies of survival and marital status is the lack of marital histories. Like other studies on this subject we used only the status at the time of diagnosis. This limitation of our study implies that we cannot, for instance, distinguish people who divorced after the diagnosis or those who became widowed during follow-up.

Our data do not allow any firm conclusion about the reason for the association between marital status and survival. We think the effect of marriage is a combination of the above-mentioned factors, and our findings may have implications for psychosocial intervention after surgery for colorectal cancer.

### References

AUVINEN A. (1992). Social class and colon cancer survival in Finland. *Cancer*, **70**, 402 – 409.

BARILLARI P, DE ANGELIS R, VALABREGA S, INDINNI MCOM, GOZZO P, RAMACCIATO G AND FEGIZ G. (1989). Relationship of symptom duration and survival in patients with colorectal carcinoma. *Eur. J. Surg. Oncol.*, **15**, 441 – 445.

BERKMAN LF AND SYME L. (1979). Social networks, host resistance, and mortality: nine-year follow-up study of Alameda County residents. *Am. J. Epidemiol.*, **109**, 186 – 204.

BLAZER DG. (1982). Social support and mortality in an elderly community population. *Am. J. Epidemiol.*, **115**, 680 – 694.

COX DR. (1972). Regression models and life tables (with discussion). *J. R. Stat. Soc. B.*, **34**, 187 – 200.

FARR W. (1859). *Influence of Marriage on the Mortality of the French People.* Savill & Edwards: London.

FOOT G, GIRGIS A, BOYLE CA AND SANSON-FISCHER RW. (1993). Solar protection behaviours: a study of beachgoers. *Aust. J. Public Heath*, **17**, 209 – 214.

GOODWIN JS, HUNT WC, KEY CR AND SAMET J. (1987). The effect of marital status on stage, treatment and survival of cancer patients. *J. Am. Med. Assoc.*, **258**, 3125 – 3130.

HOLLIDAY HW AND HARDCASTLE JD. (1979). Delay in diagnosis and treatment of symptomatic colorectal cancer. *Lancet*, **1**, 309 – 314.

HOUSE JS, ROBBINS C AND METZNER HL. (1982). The association of social relationships and activities and mortality: prospective evidence from the Tecumseh. *Am. J. Epidemiol*, **116**, 123 – 140.

INOUE M, TAJIMA K, HIROSE K, HAMAJIMA N, TAKEZAKI T, HIRAI T, KATO T AND OHNO Y. (1995). Subsite-specific risk factors for colorectal cancer: a hospital-based case – control study in Japan. *Cancer Causes Control*, **6**, 14 – 22.

JOHANSEN C, MELLEMGAARD A, SKOV T, KJAERGAARD J AND LYNGE E. (1993). Colorectal cancer in Denmark 1943 – 1988. *Int. J. Colorectal Dis.*, **8**, 42 – 47.

KATO I, TOMINAGA S AND IKARI A. (1992). The role of socioeconomic factors in the survival of patients with gastrointestinal cancers. *Jpn J. Clin. Oncol.*, **22**, 27 – 277.

KOGEVINAS M. (1990). *Longitudinal Study. Socio-demographic Differences in Cancer Survival.* Office of Population Censuses and Surveys: London.

KVIKSTAD A, VATTEN LJ AND TRETLI S. (1995). Widowhood and divorce in relation to overall survival among middle-aged Norwegian women with cancer. *Br. J. Cancer*, **71**, 1343 – 1347.

LERMAN C, RIMER BK, DALY M, LUSTBADER E, SANDS C, BALSHEM A, MASNY A AND ENGSTROM P. (1994). Recruiting high risk women into a breast cancer health promotion trial. *Cancer Epidemiol. Biomarkers Prev.*, **3**, 271 – 276.

LEVY SM, HERBERMAN RB, WHITESIDE T, SANZO K, LEE J AND KIRKWOOD J. (1990). Perceived social support and tumor estrogen/progesterone receptor status as predictors of natural killer activity in breast cancer patients. *Psychosom. Med.*, **52,** 73–85.

MACARTHUR C AND SMITH A. (1984). Factors associated with speed of diagnosis, referral and treatment in colorectal cancer. *J. Epidemiol. Community Health*, **38,** 122–126.

MELLEMGAARD A, MØLLER-JENSEN O AND LYNGE E. (1989). Cancer incidence among spouses of patients with colorectal cancer. *Int. J. Cancer*, **44,** 225–228.

NATIONAL BOARD OF HEALTH. (1986). *Classification of Disease 1965*. Copenhagen.

NEALE AV, TILLEY B AND VERNON SW. (1986). Marital status, delay in seeking treatment and survival from breast cancer. *Soc. Sci. Med.*, **23,** 305–312.

ÖSTERGREN PO. (1991). Psychosocial Resources and Health with Special Reference to Social network, Social Support and Cardiovascular Disease. Thesis. University of Malinö, Sweden.

PULLEN E, NUTBEAM D AND MOORE L. (1992). Demographic characteristics and health behaviours of consenters to medical examination. Results from the Welsh Heart Health Survey. *J. Epidemiol. Community Health*, **46,** 455–459.

RAMAZZINI B. (1965). De virginum vestalium valetudine tuenda dissertatio (a dissertation on the care of the health of nuns). *J. Occup. Med.*, **7,** 516–520.

REYNOLDS P AND KAPLAN GA. (1990). Social connections and risk for cancer: prospective evidence from the Alameda county study. *Behavior. Med.*, **Fall,** 101–110.

ROBINSON E, MOHILEVER J, ZIDAN J AND SAPIR D. (1986). Colorectal cancer: incidence, delay in diagnosis and stage of disease. *Eur. J. Cancer Clin. Oncol.*, **22,** 157–161.

SCHOENBACH VJ, KAPLAN BH AND FREDMAN L. (1986). Social ties and mortality in Evans county, Georgia. *Am. J. Epidemiol.*, **123,** 577–591.

STAVOLA BD. (1987). Statistical facts about cancers on which doctor Rigoni-Stern based his contribution to the surgeons' subgroup of the IV congress of the Italian scientists on 23 September 1842. *Stat. Med.*, **6,** 881–884.

TURUNEN MJ AND PELTOKALLIO P. (1982). Delay in the diagnosis of colorectal cancer. *Ann. Chir. Gynecol.*, **71,** 277–282.

UMBERSON D. (1992). Gender, marital status and the social control of health behavior. *Soc. Sci. Med.*, **34,** 907–917.

YI JK. (1994). Factors associated with cervical cancer screening behavior among Vietnamese women. *J. Community Health*, **19,** 189–200.