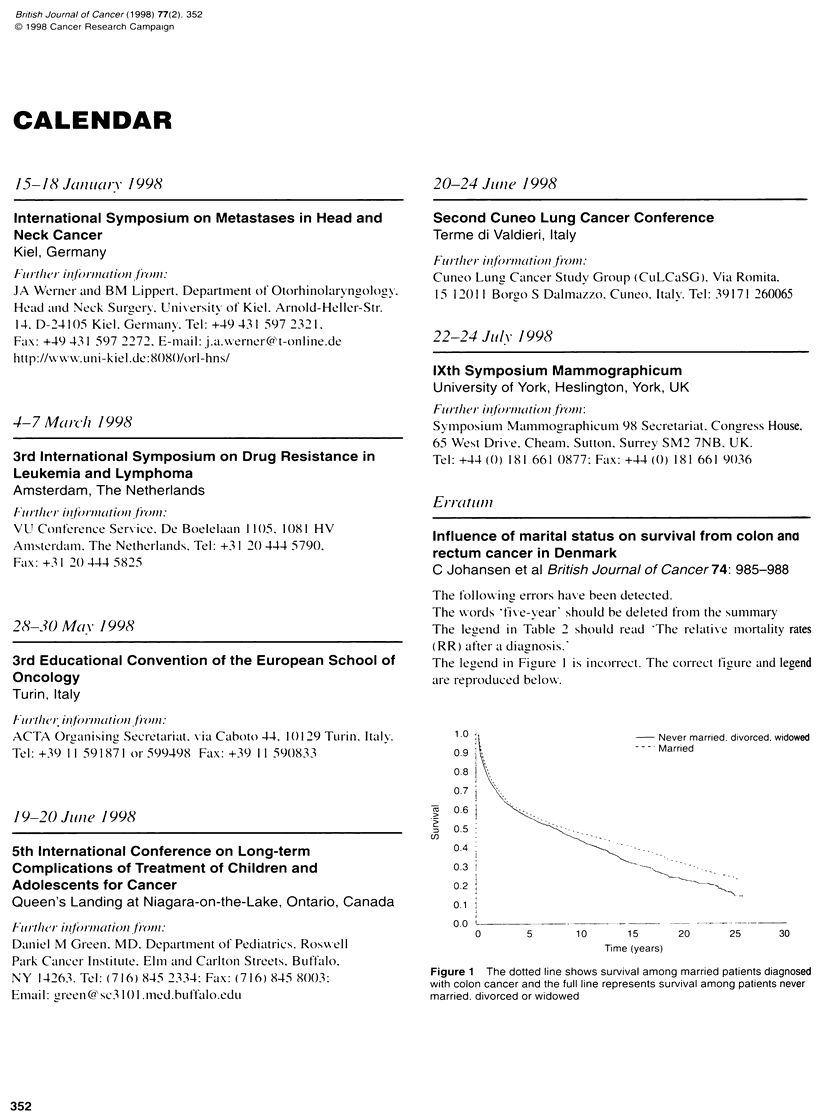# Influence of marital status on survival from colon and rectum cancer in Denmark

**Published:** 1998

**Authors:** 


					
Er-ra6ltl(i)

Influence of marital status on survival from colon anc
rectum cancer in Denmark

C Johansen et al British Journal of Cancer 74: 985-988
The followingy errors haxve been detected.

The words five-year should be deleted from the summnary

The legend in Table 2 should read 'The relative mortality rates
(RR) after a i dianosis.

The legend in Figure 1 is incorrect. The correct figure and legend
are reproduced below.

1.0                                Never married, divorced, widowed
0?9                                Married
0.8

0.7i
7   0.6

0.5
0.4
0.3
0.2
01i

0 0 o  1       .        .

0        5       10       15       20      25      30

Time (years)

Figure 1 The dotted line shows survival among married patients diagnosed
with colon cancer and the full line represents survival among patients never
married, divorced or widowed

352