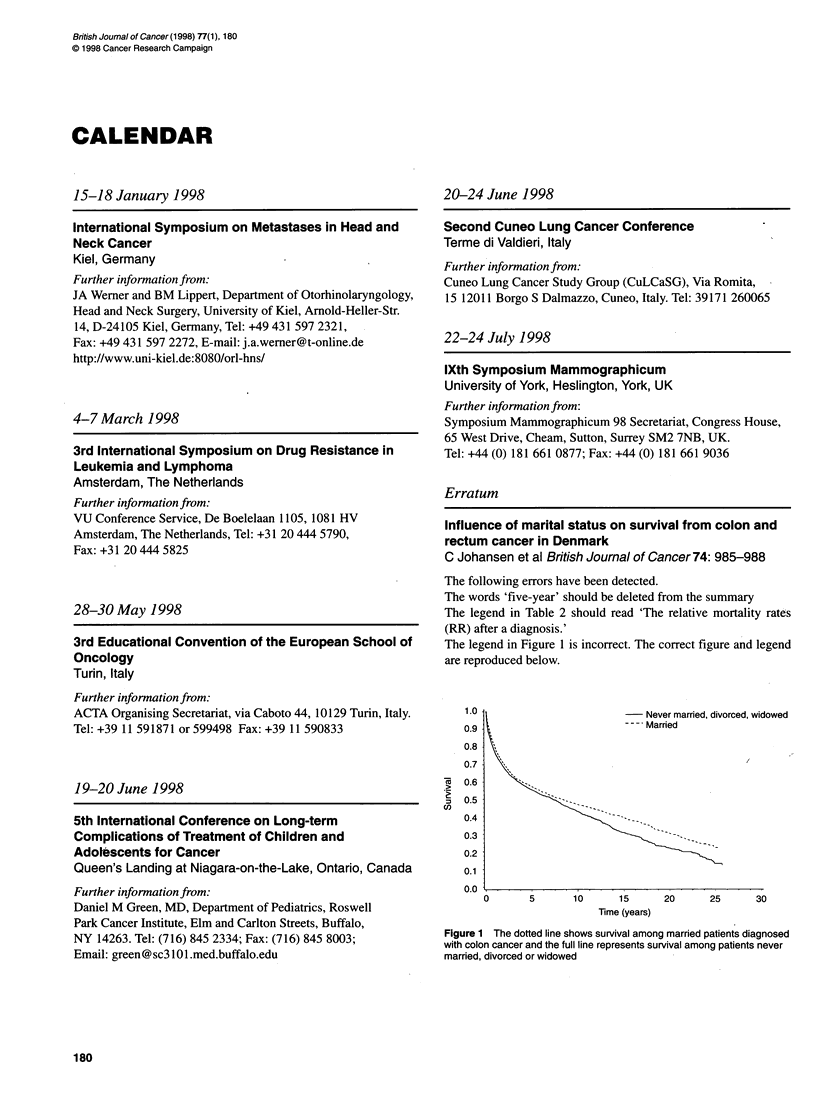# Influence of marital status on survival from colon and rectum cancer in Denmark

**Published:** 1998

**Authors:** 


### *Erratum*

**Influence of marital status on survival from colon and rectum cancer in Denmark**
C Johansen et al *British Journal of Cancer* **74**: 985–988

The following errors have been detected.
The words 'five-year' should be deleted from the summary
The legend in Table 2 should read 'The relative mortality rates (RR) after a diagnosis.'
The legend in Figure 1 is incorrect. The correct figure and legend are reproduced below.

**Figure 1** The dotted line shows survival among married patients diagnosed with colon cancer and the full line represents survival among patients never married, divorced or widowed